# Letter to the Editor Authors’ Response: Choroid and choriocapillaris changes in early-stage Parkinson’s disease: a swept-source optical coherence tomography angiography-based cross-sectional study

**DOI:** 10.1186/s13195-022-01148-8

**Published:** 2023-02-20

**Authors:** Yifan Zhang, Yuzhu Gao, Hanyue Xu, Yi Chen, Ming Zhang

**Affiliations:** 1 Department of Ophthalmology, Sichuan University West China Hospital, 37 Guoxue Lane, Wuhou District, Chengdu, 610041 Sichuan China; 2 Department of Ophthalmology, West China School of Medicine, Sichuan University, Chengdu, 610041 Sichuan China

To the Editor,

We thank Dr. Salih Uzun and Dr. Fatma Uzun for their interest regarding our recent publication entitled ‘Choroid and choriocapillaris changes in early-stage Parkinson’s disease: a swept-source optical coherence tomography angiography-based cross-sectional study’ [1]. Dr. Uzun commented on the effects of environmental factors and systemic conditions on choroidal thickness (CT) and wished to understand how we considered these factors in our study. We would like to take this opportunity to respond to several points mentioned in their letter.

First, as we mentioned in the “Discussion” section of our article, we completely agreed with Dr. Uzun that the choroid is a dynamic vascular tissue that can be affected by intrinsic and extrinsic factors [2]. Knowing this, our study, a matched case-control study, was designed to eliminate the effects of previously reported influencing factors. Baseline demographics including gender, age, spherical equivalent (SE), best-corrected visual acuity (BCVA), intraocular pressure (IOP), smoking status, and alcohol use exhibited no significant differences among the Parkinson’s disease (PD) group and control group. All of our study results were also consistent after adjusting for these factors.

Second, all participants were enrolled based on strict inclusion and exclusion criteria. Patients in the PD group were free of any systemic or ophthalmic diseases other than PD. And participants in the control group were age- and gender-matched healthy subjects without any systemic or ophthalmic conditions. Please refer to the “Method” section of our article for the detailed inclusion and exclusion criteria for more information [1]. As far as the other variables mentioned in Dr. Uzun’s letter, some of them were not applicable in our cohort of patients. For example, Dr. Uzun commented on whether menstrual phases and pregnancy status were taken into consideration in our study. PD is a neurodegenerative disease that mainly occurred in the elderly population [3]. Upon enrollment, all female participants in our study were post-menopausal, and none of them were pregnant at the time of the study. Therefore, the effects of those two factors on choroidal measurements were strictly eliminated from our study. We were also aware that caffeine consumption and exercise were reported to be related to CT changes [4, 5]. However, we found the results of those studies contradicting. Also, it is fairly rare for the elderly population in our local area to consume caffeine beverages based on our understanding of the cohort from a cultural perspective. Therefore, we did not record information regarding caffeine intake in our study. As far as exercise, before the capturing of SS-OCTA images, all participants underwent a full neuro-ophthalmic evaluation in a quiet interviewing room in a sitting position. No participants underwent violent aerobic or anaerobic exercise before the evaluation and obtaining SS-OCTA images, which abolishes the short-term effects of exercise on CT.

Third, the diurnal modulation of the choroidal circulation has been widely reported, which was reported to be the thickest at midnight and the thinnest at noon [6, 7]. Therefore, we conducted our study between 9 am and 12 pm to minimize the influence of diurnal variation of the choroid on our study, which is also a widely practiced protocol for many other choroidal circulation studies.

To conclude, we would like to thank Dr. Uzun and his team again for their letter and their contribution to our study. Although various variables have effects on CT, this matched case-control study adopted strict inclusion and exclusion criteria to abolish the influence of reported influencing factors on the outcome. Our SS-OCTA images were collected within a narrow time window to minimize the effects of diurnal modulation. The study design and consideration for individual influencing factors contribute to the reliability of our study results.

## Data Availability

Not applicable.
